# Gorlin-Goltz Syndrome

**DOI:** 10.1155/2012/247239

**Published:** 2012-10-03

**Authors:** Padma Pandeshwar, K. Jayanthi, D. Mahesh

**Affiliations:** ^1^Sri Venkateshwara Dental College and Hospital, Kariyappanahalli, Anekal Road, Bannerughatta, Bangalore 560083, India; ^2^Oral Medicine, Diagnosis and Radiology, Bangalore Institute of Dental Sciences, Lakkasandra, Wilson Garden, Bangalore, India; ^3^Dayananda Sagar College of Dental Sciences, Shivage Malleshwara Hills, Kumarswamy Layout, Bangalore, India

## Abstract

The Gorlin-Goltz syndrome (GGS) (the nevoid basal cell carcinoma syndrome—NBCCS) is a rare autosomal dominant syndrome caused due to mutations in the *PTCH* (patched) gene found on chromosome arm 9q. The syndrome, characterized by increased predisposition to develop basal cell carcinoma and associated multiorgan anomalies, has a high level of penetrance and variable expressiveness. GGS is a multidisciplinary problem, early diagnosis of which allows introduction of secondary prophylaxis and following an appropriate treatment to delay the progress of the syndrome. The following report emphasizes the need for awareness of the diagnostic criteria of this syndrome in cases with no typical skin lesions.

## 1. Introduction

GGS, also known as nevoid basal cell carcinoma syndrome (NBCCS), is an infrequent multisystemic disease with an autosomal dominant trait, with a complete penetrance and variable expressivity, though sporadic cases have been described [1, 2]. GGS shows a predisposition to neoplasms and other developmental abnormalities. The estimated prevalence varies from 1/57,000 to 1/256,000 among various studies, with a male-to-female ratio of 1 : 1 [2].

The first report of the syndrome was made in 1894 by Jarisch and White in a patient with multiple basal cell carcinomas, scoliosis, and learning disability. Binkley and Johnson in 1951, and Howell and Caro in 1959 suggested a relationship between basal cell epitheliomas and developmental malformations. It was delineated only in 1960 by Robert James Gorlin and William Goltz [3, 4] who established the classical triad (multiple basocellular epitheliomas, keratocysts in the jaws and bifid ribs) that characterizes the diagnosis of this syndrome. This triad was later modified by Rayner et al., who established that the diagnostic criteria would require cysts to appear in combination with calcification of the falx cerebri or palmar and plantar pits [5–7].

In addition to the classical triad described by Gorlin and Goltz, calcification of the falx cerebri, palmar and plantar epidermal pits, spine and rib anomalies, relative macrocephaly, facial milia, frontal bossing, ocular malformation, medulloblastomas, cleft lip and/or palate, and developmental malformations have also been established as features of the syndrome [5–7].

The pathogenesis of GGS is attributed to be the consequence of abnormalities in the *PTCH* gene. The loss of human patched gene (PTCH1 gene), a tumor suppressor gene, forms the molecular basis of the syndrome [8]. This gene is significant for embryonic structuring and cellular cycle, thus its mutation leads to the development of the disease including neoplasms. The syndrome exhibits abnormalities similar to those seen in people exposed for long periods to UV radiation. Several different mutations of the PTCH1 gene have also been identified in patients with GGS [2, 3]. 

It is important to establish an earlier diagnosis to prevent fatal consequences, due to multiple skin cancers and other tumors associated with the syndrome [8]. Furthermore, our case emphasizes the role of the dentist in recognizing these features in order to arrive at an early diagnosis and a multidisciplinary approach in treating the condition.

## 2. Case Report

A 38-year-old male patient reported to the OPD of our department with a chief compliant of swelling in the right lower back tooth region since 3 months and gave a history of extraction with respect to 46 and 47 (carious teeth) 7 months ago. This was followed by persistent pus discharge from the region of extraction. Three months back he was referred to a hospital but was refused treatment owing to medical risk (Asthmatic). In the meanwhile the swelling had not increased in size and had no associated pain or discomfort. There was h/o regular discharge of creamy viscous fluid from the gingival sulcus of adjacent teeth.

**Figure 1:**
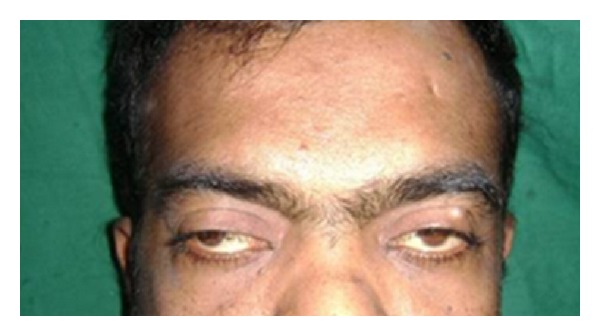
Broad nasal bridge.

**Figure 2:**
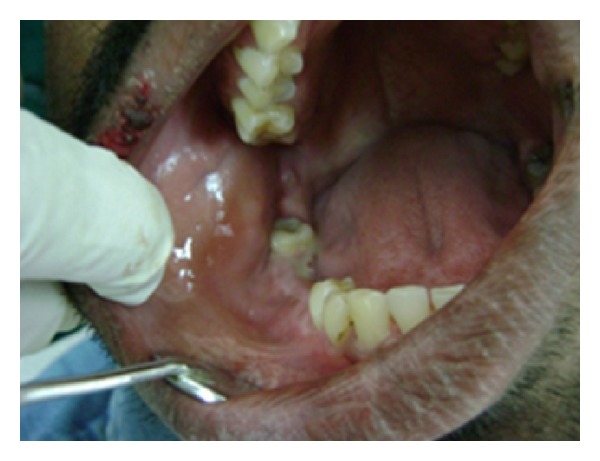
Intraoral swelling in relation to 46, 45.

 Patient was a known case of asthma for which he was undergoing treatment. Patient was asked for CVS, hematologic, neurological abnormalities, and for allergies with no relevant history. Personal history was insignificant, but family history revealed his 9-year-old daughter had similar bilateral mandibular swelling, but no further investigations had been done. Extraoral examination revealed an increased fronto-occipital circumference of the head (57 cm), frontal bossing, hypertelorism, and strabismus with the right eye (Figure 1). Intraoral examination of the right posterior mandibular region in relation to 44, 45, and 46 showed a diffuse solitary swelling measuring 2 × 4 cm in size which was soft and fluctuant in consistency (Figure 2).

**Figure 3:**
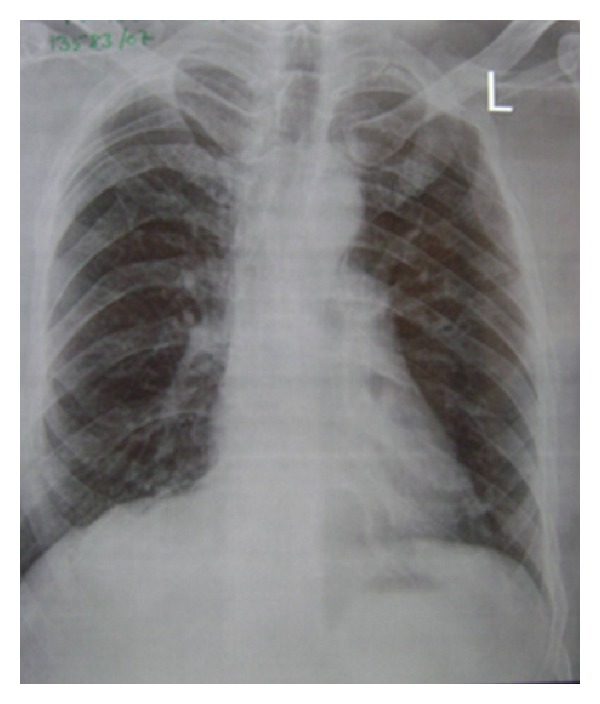
Bifid rib right 4th and 8th rib anteriorly.

**Figure 4:**
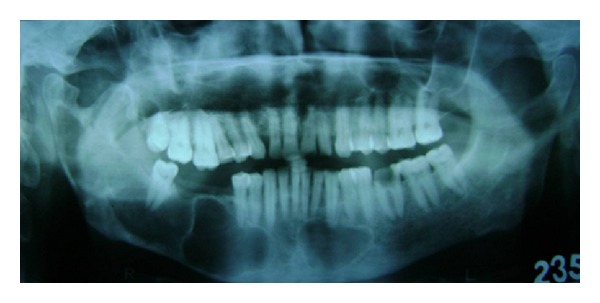
Panoramic radiograph showing cystic lesions in the mandible.

**Figure 5:**
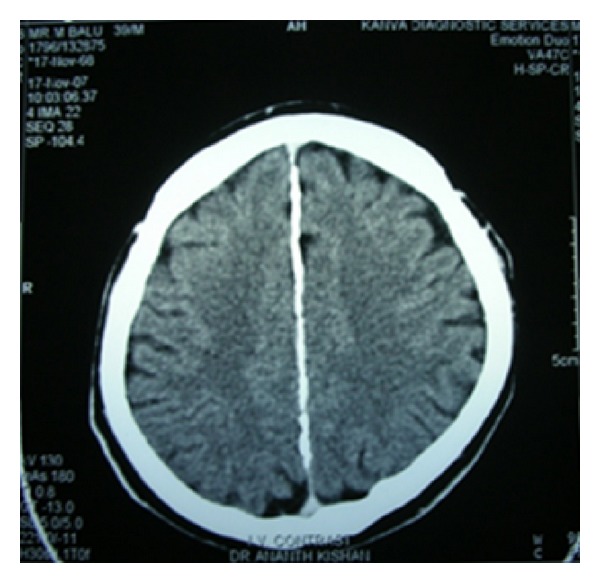
Axial CT of brain showing calcification of falx cerebri.

**Figure 6:**
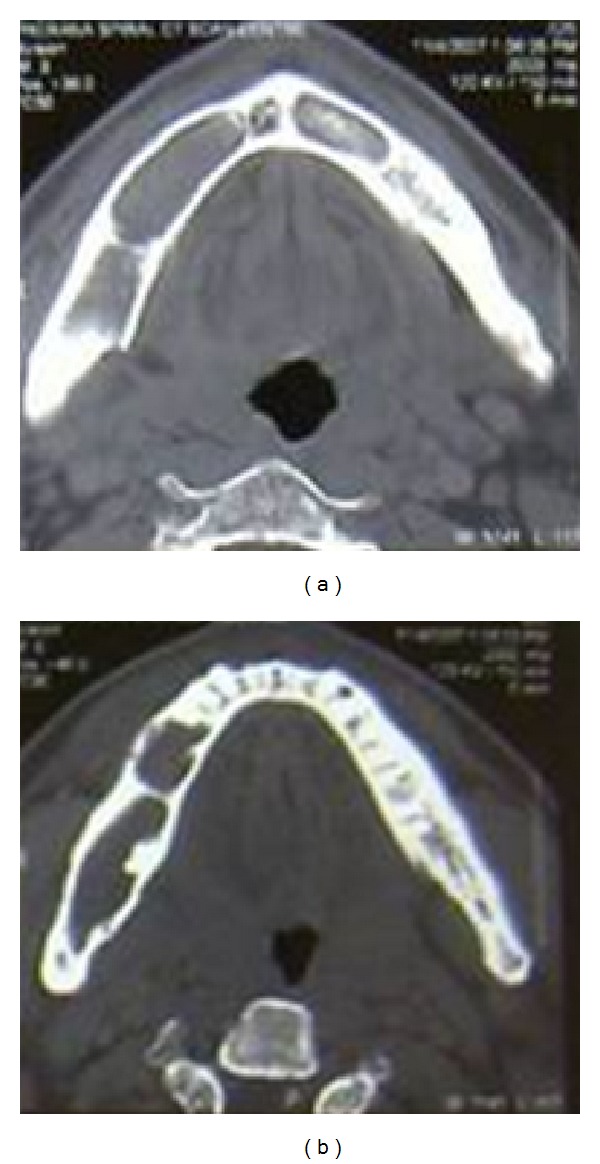
CT scan showing cystic lesions of body and condyle of the mandible.

 On OPG 3 cystic lesions were seen in the mandible with the apical regions of 33, 34, 35, 44, 45, and 48. Both the condylar and coronoid processes on the right side were deformed in comparison to those of the left side (Figure 4). The maxillary alveolar bone in relation to the apical regions of 14, 15, and 16 showed a diffuse area of mixed radiolucent and radiopaque lesion with ground glass appearance. Chest radiograph showed bifid right 4th and 8th rib anteriorly (normal variant) (Figure 3). CT scan of the brain showed lamellar calcification along the falx and tentorium (Figure 5). A mottled appearance in the skull vault was seen in the parietal region bilaterally. CT scan of the face revealed an enlargement of the right mandible with two osteolytic lesions (Figure 6) and ground glass appearance of the marrow with calcific densities within. Aspiration was performed with the mandibular lesion and showed a cheesy fluid which was sent for histopathological evaluation.

Based on the patient's history, clinical findings, and radiological findings a provision diagnosis of Gorlin-Goltz syndrome was given. Differential diagnosis of Bazex Syndrome and Torre's syndrome was given. The patient later underwent enucleation of the cystic lesions of both the maxilla and the mandible, with primary closure. The specimens were sent for histopathological evaluation which confirmed the diagnosis of multiple odontogenic cysts. 

 The final diagnosis of Gorlin-Goltz syndrome with fibrous dysplasia of the skull bones was reached.

## 3. Discussion

In the case of GGS it is important to make an early diagnosis as these patients show increased propensity to multiple malignant neoplasms and are also sensitive to ionizing radiation including UV radiation [3]. Patients' undergoing regular and detailed checkups can reduce the severity of complications, such as malignant skin, brain tumors, and maxillofacial deformities due to odontogenic keratocysts.

Diagnosis is based upon established major and minor clinical and radiological criteria and is ideally confirmed by DNA analysis [8]. The diagnostic criteria for nevoid basal cell carcinoma, established by Evans et al. and modified by Kimonis et al. in 1997, state that when two major or one major and two minor criteria should be present for diagnosis, as described below [3–9].

**(I) Major Criteria**

- More than two basal cell carcinomas or one basal cell carcinoma at younger than 30 years of age or more than 10 basal cell nevi.
- Any odontogenic keratocyst (proven on histology) or polyostotic bone cyst.
- Three or more palmar or plantar pits (present in about 65% of patients).
- Bifid, fused, or markedly splayed ribs.
- Ectopic calcification: lamellar or early at younger than 20 years of age.
- Falx cerebri calcification.
- Positive family history of nevoid basal cell carcinoma.

Some authors take plurilamellar appearance of the falx cerebri calcification as a pathognomonic symptom of Gorlin-Goltz syndrome.

**(II) Minor Criteria**

- Macrocephaly determined after adjustment with height.
- Skeletal anomalies: hemivertebrae, scoliosis, syndactyly, polydactyly, and shortened 4th metacarpal.
- Radiological abnormalities like bridging of sella turcica, vertebral anomalies, and modelling defect of hands and feet.
- Medulloblastoma.
- Ovarian Fibroma.
- Congenital malformations: cleft lip or palate, polydactylism or eye anomalies (cataract, coloboma, and microphthalmus).

In the above case, two major criteria (odontogenic keratocysts of the jaw and calcification of falx cerebri and tentorium) and one minor (skeletal anomalies (bifid rib)) were detected, suggesting that the patient had GGS.

Appropriate management depends on early recognition of the disease, a detailed family history, and a thorough evaluation of signs and symptoms. A multidisciplinary approach team consisting of various specialists is required for a successful treatment.

 Treatment involves removal of tumors by surgical excision, laser ablation, photodynamic therapy, or topical chemotherapy, while radiotherapy is a contraindication. Chemoprevention involves use of vitamin A analogs. Recurrent odontogenic cysts (up to 60% of cases) require repeated surgical excisions. 5–10% of the patients may develop brain medulloblastoma, a potential cause of early death, thus requiring intervention by a neurologist. Though survival in Gorlin-Goltz patients is not affected significantly, morbidity from complications can be considerable. Nowadays gene mutation analysis, if feasible, can confirm diagnosis. Antenatal diagnosis is possible with ultrasound scans and DNA analysis. Thus, a genetic counsellor is of importance in the ongoing care of the patient [8, 10].

A new treatment strategy, based on the understanding of the Hh signaling pathway and the premise that tumors arise due to its overactivity, supposes that inhibition of this pathway with specific pharmacological treatment might suppress tumor growth [3, 11].

## 4. Conclusion

The case illustrates the need for awareness of the syndrome among dentists in relation to younger age patients with no lesions of the skin. Proper evaluation and characterization of clinical features are essential for the correct diagnosis and management. Ongoing surveillance as well as treatment for sequelae of Gorlin-Goltz syndrome (GGS) requires regular followups (3-4 times a year or more) to detect new odontogenic cysts and basal cell carcinomas that occur continuously [8, 10, 11]. This, along with genetic counseling in family members of patients with GGS, as in the above case who has a daughter with related symptoms, in whom the diagnosis is possible but not confirmed, helps to detect necessary diagnostic criteria and thus improves their survival through well-directed treatment.
